# Facilitated Subcutaneous Immunoglobulin Replacement Therapy in Clinical Practice: A Two Center, Long-Term Retrospective Observation in Adults With Primary Immunodeficiencies

**DOI:** 10.3389/fimmu.2020.00981

**Published:** 2020-05-20

**Authors:** Ewa Wiesik-Szewczyk, Dariusz Sołdacki, Leszek Paczek, Karina Jahnz-Różyk

**Affiliations:** ^1^Department of Internal Medicine, Pulmonology, Allergy and Clinical Immunology, Central Clinical Hospital of the Ministry of National Defense, Military Institute of Medicine in Warsaw, Warsaw, Poland; ^2^Department of Clinical Immunology, Medical University of Warsaw, Warsaw, Poland; ^3^Department of Immunology, Transplantology and Internal Diseases, Medical University of Warsaw, Warsaw, Poland

**Keywords:** common variable immunodeficiency, hypogammaglobulinemia, fSCIG, personalized approach, pregnancy, primary immunodeficiency, replacement immunoglobulin therapy, real-life data

## Abstract

Facilitated subcutaneous immunoglobulin (fSCIG) replacement therapy is the latest method of IgG administration; however, real-life data are limited. We retrospectively analyzed the everyday experience of fSCIG administration, particularly, the method used to switch from intravenous immunoglobulin (IVIG) or subcutaneous immunoglobulin (SCIG) to fSCIG and the dosing modifications required. Of the 39 adult patients with primary immunodeficiency (PID) who received fSCIG, 34 remained on the therapy at the end of the study. The median observation time was 18 (range, 3–24) months. Two patients were IgG-treatment-naïve; 23 had previously received IVIG and 14 had received SCIG. In 25 cases, a non-ramp-up dosing mode was used to switch to fSCIG (including two half-monthly doses given biweekly in 14 cases, and full monthly doses given in 11 cases), a ramp-up mode was used in six cases; other methods were used in eight cases. The median IgG trough level at baseline was 7.9 g/L (*n* = 38), 7.9 g/L (*n* = 32) at Month 6, 9.0 g/L (*n* = 30) at Month 12, 8.6 g/L (*n* = 22) at Month 18, and 9.0 g/L (*n* = 11) at Month 24. No serious bacterial infections or hospitalizations due to PID complications occurred. At the end of the study, 24 patients (71%) received fSCIG every 4 weeks, six (18%) received fSCIG every 3 weeks, and four (12%) received fSCIG biweekly. In conclusion, our study provides real-life evidence of clinical efficacy of personalized fSCIG treatment when switching from prior immunoglobulin replacement using various switching modes and dosing frequencies.

## Introduction

Immunoglobulin G (IgG) replacement therapy is the most essential pharmacological intervention in patients with humoral primary immunodeficiency (PID). The substitution of antibodies significantly reduces mortality as it prevents patients from many serious health conditions, most commonly, recurrent bacterial infections (1). Due to the primary character of the immunity defect, the treatment must be systematically conducted throughout ones' lifetime, either intravenously (IVIG) or subcutaneously (SCIG). Two methods of SCIG application are currently available, termed conventional (either using an infusion pump or without, termed rapid-push SCIG) and facilitated (fSCIG), which is aided by the initial administration of human recombinant hyaluronidase in the same needle as IgG.

Home SCIG is safe, clinically-effective, cost-effective, and often preferred by patients and medical staff (2, 3). However, conventional SCIG can be burdensome due to the high frequency of infusions required (i.e., weekly dosing) (4). Weekly dosing is required in conventional SCIG, as only a limited volume of IgG can be infused into the subcutaneous tissue. Meanwhile, the fSCIG method allows a larger volume of IgG to be administered, and therefore, only requires dosing every 4 weeks, analogous to IVIG (5). This may reduce the burden of SCIG treatment, as well as improve patients' quality of life and their adherence to treatment. Therefore, the fSCIG method can fulfill patients' expectations; i.e., it involves home-based 4-weekly infusions that can be self-administrated with shorter administration and fewer needle sticks (4).

The extensions of pivotal clinical studies have shown fSCIG is an effective and safe option both for adults and children with PID (6, 7). However, data from real-life experience, especially regarding practical aspects of switching patients to fSCIG, remain limited. Indeed, most data has been derived from case reports (8–12), with only one single-site, real-life study published in 2014 (13). Unfortunately, this real-life study was limited to only 14 patients, with a short follow-up (8 months), and a fixed dosing schedule (every 3 weeks) (13). Moreover, in terms of switching from other types of IgG replacement to fSCIG, available data is limited to the ramp-up dosing mode.

In this long-term, retrospective, open observational study, we aimed to report the everyday experience of fSCIG treatment in adult patients with PID. In particular, we examined the reasons for switching, the mode of switching, and the modification of dosing (according to patients' expectations of the planned 4-weekly application and the shared-decision making model) in a long-term follow-up in routine clinical practice.

## Materials and Methods

This was a retrospective analysis of routinely-collected, real-life data obtained from the medical documentation of PID patients receiving fSCIG treatment. The patient database was closed on June 30, 2019. The study started on January 24, 2017, which was the day of the first administration of fSCIG therapy.

Patients eligible to participate in the study were: adults (aged ≥18 years), with humoral PID diagnosed according to the European Society for Immunodeficiencies (ESID) criteria (14), who were receiving fSCIG substitution treatment covered by the Drug Program no B.62 financed by the National Health Fund in Poland (15). Patients were treated in two Immunology Centers specialized in PID therapy located in Warsaw, Poland. In both centers, all modes of IgG administration (IVIG, SCIG, and fSCIG) and all licensed immunoglobulin products were available. After completing an educational period, patients continued self-treatment in a home-based manner and were controlled every 3 months.

We collected demographic data and data on the everyday experience of fSCIG administration. This included the reasons for switching from IVIG or SCIG to fSCIG, as well as the practical way the method of administration and dose was changed according to the patients' expectations in the context of the 4-weekly application and the infusion characteristics (i.e., number of administration sites, flow characteristics, and volume of the administered drug). As the shared-decision model of care is applied in the two treatment centers (16), it was possible to report the reasons for changing the therapy. During routine control visits, the professional medical staff (physicians and nurses) assessed treatment effectiveness in terms of clinical response, treatment compliance, and patient's subjective evaluation (i.e., patient satisfaction, burden of the treatment). Based on the assessment and presentation of the available alternative methods of therapy, the decision regarding the next course of treatment was made in the cooperation with the patient.

The ramp-up mode of switching is described in the registration clinical trial protocol (6) and recommended in the HyQvia Summary of Product Characteristics (17) as follows: 25% of the dose infused at Week 1, 50% at Week 2, 75% at Week 4, and 100% at Week 7.

The fSCIG monthly dose was calculated based on the individual's demand over the past 3 months, independent of the previous mode of administration, and then corrected according to the patient's needs (18). The aim of introducing fSCIG therapy is to obtain the individual IgG trough level that provides optimal protection from infectious diseases. Lucas et al. showed a wide range of IgG trough levels and drug doses may be used to control infections (19). Here we aimed to achieve a trough IgG level of >5.0 g/L (20) that optimally reduced infection rates in the particular patient and was preferably within the reference range of the local laboratory (i.e., 7.0–12.0 g/L). In assessing patient's needs, we also took into account their subjective overall well-being and symptoms related to wear-off effect and fatigue (21).

To assess efficacy, we determined IgG trough levels (drawn just before the next infusion) and the proportion of patients with serious bacterial infections or hospitalizations due to PID complications. A serious bacterial infection was defined as bacterial pneumonia, bacteremia/septicemia, osteomyelitis/septic arthritis, bacterial meningitis, and visceral abscess (22). One patient with hyperglobulinemia at baseline, was excluded from IgG trough level analysis.

In this study, mainly descriptive statistical methods were used. For continuous variables, we calculated the median and range; and for categorical variables, we determined the frequency counts and percentages. The significance of changes in the IgG trough levels was assessed using the Wilcoxon signed-rank test. The significance of differences between infused volumes of IgG in patients receiving different dosing intervals was assessed using the multiple comparisons (two-tailed) Kruskal-Wallis test. All computations were done using Microsoft Excel software and TIBCO Software Inc. Statistica ver. 13.

The Bioethics Committee of the Military Institute of Medicine in Warsaw approved the study (Approval No. 55/WIM/2019). All patients gave written consent for the treatment and inclusion of their medical and treatment history records within this study.

## Results

At the time the database was closed, there were 39 adult patients with PID that had received fSCIG replacement therapy (median observation time of 18 months; range, 3–24 months). The first patient started fSCIG in January 2017. Prior to fSCIG treatment, two patients were naïve to any immunoglobulin replacement therapy (IgRT). Among those that had received prior IgRT, 23 received IVIG, five self-administered SCIG, and nine received both IVIG and SCIG. The median time of prior IgRT (both IVIG and SCIG) for the 37 patients was 38 (range, 1–252) months. The detailed characteristics of the included patients are presented in (Table 1).

**Table 1:** Patient characteristics.

**Characteristic**	All study participants (*N* = 39)^*^
**Age**, median (range), years	35 (18–68)
**Sex**	
Female	21 (54%)
Male	18 (46%)
**Type of primary immunodeficiency**	
Common variable immunodeficiency Unspecified hypogammaglobulinemia X-linked or recessive agammaglobulinemia Antibody deficiency due to congenital defect Specific antibody deficiency with bronchiectasis Hyperglobulinemia with infections	27 (71%) 5 (13%) 3 (8%) 2 (5%) 1 (3%) 1 (3%)
**Disease duration**, median (range), years	10 (1–41)
**Place of living**	
Big city Small city Village	25 (64%) 11 (28%) 3 (8%)
**Education level**	
High Secondary Basic	18 (46%) 14 (36%) 7 (18%)
**Professional activity**	
Professionally active (working/studying) Disability benefit	24 (62%) 15 (38%)
**IgG replacement therapy before fSCIG**	
Only IVIG IVIG>SCIG Only SCIG IgG naïve	23 (59%) 9 (23%) 5 (13%) 2 (5%)

* *All data are presented as n (%), unless otherwise indicated*.

*fSCIG, facilitated subcutaneous immunoglobulin; IVIG, intravenous immunoglobulin; SCIG, subcutaneous immunoglobulin*.

The median number of fSCIG infusions per patient was 26 (*N* = 34; range, 6–59). Due to different treatment durations, data were available for 32 patients in Month 6 of fSCIG therapy, 30 patients in Month 12, and 22 patients in Month 18. Eleven patients received fSCIG for 24 months. Overall 894 infusions were analyzed in this study. At the time the database was closed, 34 patients were still on fSCIG therapy. Five patients discontinued fSCIG for the following reasons: two male patients returned to 20% conventional SCIG due to side effects after the first fSCIG application; one woman with unspecified hypogammaglobulinemia switched to rapid-push application of 20% L-proline-stabilized IgG on the second educational visit at her request (she had problems programming the pump and preferred to apply smaller volumes of the drug at one site), and; two women were non-compliant (i.e., they gave up any type of IgRT and were lost from follow up; one in the 12th week of observation, and the other in week 36th). One patient with hyperglobulinemia at baseline, was excluded from IgG trough level analysis.

### Reasons for Switching to fSCIG

The reasons for switching to fSCIG therapy were patient preference (28 patients, 72%), medical reasons (four patients, 10%), or both (seven patients, 18%). Medical reasons included: headaches or flu-like syndromes after IVIG (four cases), difficult or lack of venous access (five cases), severe common variable immune deficiency (CVID) enteropathy combined with IVIG insufficiency (one case), lack of adherence to conventional SCIG (one case).

### Treatment Scheme and Dosing

The following methods of introducing fSCIG therapy were used: non-ramp-up dosing in 25 patients (i.e., two initial half-monthly doses biweekly in 14 patients, and full monthly dosing in 11 patients), ramp-up dosing in six patients, and other methods that were modified according to patient's preferences and center accessibility in eight patients.

At the time the database closed, 34 patients were continuing fSCIG treatment: 24 patients (71%) received fSCIG every 4 weeks, six patients (18%) every 3 weeks and, four patients (12%) every 2 weeks. The main reasons for changing from monthly to more frequent dosing were as follows: wear-off effect (six cases), large dose and local swelling that persisted longer than 3 days (three cases), pregnancy (two cases), low trough IgG level despite the high dose of 800 mg/kg/4 weeks (one case).

According to diagnosis, patients who needed biweekly dosing were: one patient with hypogammaglobulinemia and myopathy, one patient with combined immunodeficiency and five patients with complicated CVID (i.e., three cases with polyclonal lymphadenopathy, three cases with autoimmune disorders, and two cases with bronchiectasis). Please note that more than one condition could occur in one patient.

The median initial fSCIG dose was 480 mg/kg/4 weeks (*N* = 33; range, 260–800 mg/kg/4 weeks). The median fSCIG dose was 510 mg/kg/4 weeks (*N* = 32; range, 260–800 mg/kg/4 weeks) at Month 6, 560 mg/kg/4 weeks (*N* = 30; range, 260–800 mg/kg/4 weeks) at Month 12, 505 mg/kg/4 weeks (*N* = 22; range 350–770 mg/kg/4 weeks) at Month 18, and 470 mg/kg/4 weeks (*N* = 11; range, 370–590 mg/kg/4 weeks) at Month 24.

### Infusion Characteristics

In the majority of patients (*n* = 32, 94%), one application site was used, while two patients used two application sites. The median of volume infused per site was 300 ml (*N* = 33; range 150–500 ml). No statistically significant changes were found between the median infused volumes of the drug in the groups defined by different dosing intervals (i.e., every 2, 3, or 4 weeks; *p* = 0.317; Supplementary Figure 1). Three patients used syringe pumps (CRONO S-PID 100 or Syringe Pump T34L) and 31 used infusion pumps (Bodyguard 323 Color) with the median infusion speed of 300 ml/h (*N* = 33; range, 250–300 ml/h).

### Clinical Efficacy and IgG Trough Level

There were no cases of serious bacterial infection or hospitalization due to a PID complication in our study.

**Figure 1:**
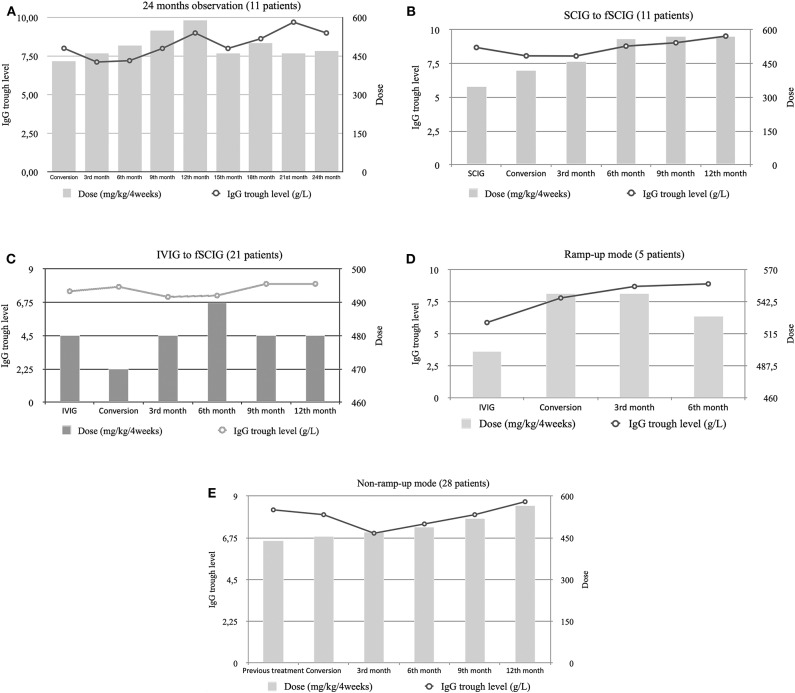
Median trough immunoglobulin G (IgG) levels and median dose per 4 weeks of immunoglobulin therapy in: **(A)** patients that completed the 24-month follow-up, **(B)** patients that switched from subcutaneous immunoglobulin (SCIG) to fSCIG, **(C)** patients that switched from intravenous immunoglobulin (IVIG) to fSCIG, **(D)** patients that switched in a ramp-up mode, and **(E)** patients that switched in a non-ramp-up mode.

The IgG trough levels and therapeutic IgG doses are summarized in (Table 2). Data for a 24-month observation period were available for 11 patients. There was a systematic growth in the median IgG trough level starting from 8.01 g/L at baseline and reaching 9.0 g/L at Month 24 (Figure 1A). A statistically significant difference was found between median IgG through levels between baseline and Month 12 (*p* = 0.024) in the whole study population (Supplementary Figure 2).

**Table 2:** Trough IgG levels and dosing (both presented as median).

	**24 months of observation** ***n*** **=** **11**		**SCIG to fSCIG** ***n*** **=11**		**IVIG to fSCIG** ***n*** **=** **21**		**Ramp-up** ***n*** **=** **5**		**Non-Ramp-up** ***n*** **=** **28**	
	**IgG** **g/L**	**Dosing** **mg/kg/4 weeks**	**IgG** **g/L**	**Dosing** **mg/kg/4 weeks**	**IgG** **g/L**	**Dosing** **mg/kg/4 weeks**	**IgG** **g/L**	**Dosing** **mg/kg/4 weeks**	**IgG** **g/L**	**Dosing** **mg/kg/4 weeks**
Baseline	8.01	430	8.07	420	7.8	470	7.8	550	8.0	455
Month 3	7.12	460	8.06	460	7.12	480	8.7	550	7.0	470
Month 6	7.21	490	8.79	560	7.21	490	8.9	530	7.5	490
Month 12	9.0	590	9.53	570	8.0	480	9.8	470	8.7	565
Month 18	8.63	500	Nd^*^	Nd^*^	Nd^*^	Nd^*^	Nd^*^	Nd^*^	Nd^*^	Nd^*^
Month 24	9.0	470	Nd^*^	Nd^*^	Nd^*^	Nd^*^	Nd^*^	Nd^*^	Nd^*^	Nd^*^

* *Nd, no data*.

*fSCIG, facilitated subcutaneous immunoglobulin; IVIG, intravenous immunoglobulin; SCIG, subcutaneous immunoglobulin*.

Among the group of patients converting from SCIG to fSCIG (*n* = 11), higher fSCIG doses were required to maintain the same IgG trough levels (Figure 1B). Meanwhile, patients previously treated with IVIG (*n* = 21) required comparable doses of fSCIG to maintain their IgG trough levels (Figure 1C). Either ramping-up the therapy dose (Figure 1D), or using a non-ramp-up method (Figure 1E), could maintain clinical efficacy.

**Figure 2:**
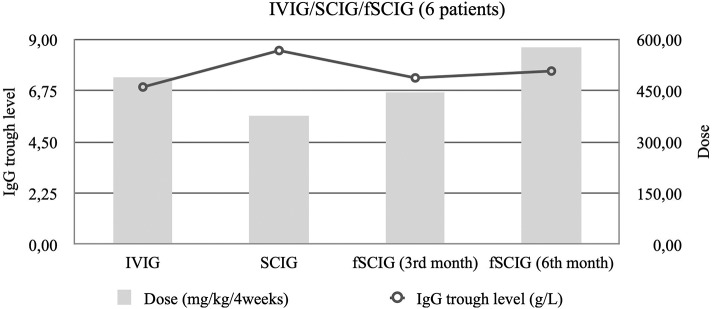
Median trough immunoglobulin G (IgG) levels and median immunoglobulin dose per 4 weeks among six patients that started of facilitated subcutaneous immunoglobulin (fSCIG) treatment after previous immunoglobulin (SCIG) and intravenous immunoglobulin (IVIG).

The trough IgG levels and doses of six patients previously treated with IVIG and then SCIG, who then switched to fSCIG were analyzed separately. These patients required doses analogous to their prior IVIG therapy, and not their immediately-preceding SCIG dose, to maintain clinical efficacy when switching to fSCIG (Figure 2).

### Tolerability

No serious adverse drug reactions were observed in our study. There was one type of local side effect (edema at the infusion site) observed in all patients and lasting for a median time of 24 (*N* = 34; range, 12–72) h. The duration of the swelling was independent of the patient's body mass index.

In addition, general side effects were observed in seven patients; among them, two decided to discontinue fSCIG. The first patient (who was previously on IVIG without any side effects) complained of low-grade fever, chills, fatigue, and edema with redness close to the site of infusion for 72 h after the first fSCIG application, after which the patient switched to 20% conventional SCIG (which was well-tolerated without side effects). The second patient (who was previously on IVIG and then 16% SCIG) reported chills, low grade fever, anxiety, a prickling sensation and palm rash after the first infusion of 12.5 g fSCIG; however, the symptoms disappeared spontaneously within 12 h. Nonetheless, after the first fSCIG infusion, the patient decided to switch to 20% conventional SCIG (which was well-tolerated long-term, without side effects). Another three patients complained of low-grade fever, chills, and fatigue appearing 24 h after the fSCIG application. All three patients had received IVIG or SCIG prior, without any side effects. In two patients, the symptoms disappeared within 3 months, and in one male patient, a sub-febrile condition persisted until the last follow-up visit at 24 months.

## Discussion

Long-term observations from clinical trials have proven the effectiveness and safety of fSCIG both in pediatric and adult PID patients (6, 7). The switch method from IVIG to fSCIG proposed in the clinical setting was a ramp-up model, with a final frequency of injections every 3–4 weeks. Although clinical data has confirmed the efficacy and safety of fSCIG, here we focus on real-life observations, which provide useful information on heterogenous populations and real-life treatment patterns, and may reveal rare adverse events (23).

Indeed, only one other real-life observation focusing on the practical aspects of everyday fSCIG experience has been conducted (13), albeit with some limitations (i.e., small sample size, short follow-up, and a fixed dosing schedule), which were improved in the methodology of our study. Ponsford et al. (13) reported identified two types of patients choosing fSCIG over IVIG or SCIG: those with clinical problems on current treatment and those looking for convenience and flexibility (13). However, we found patient preference was the main reason for switching to fSCIG.

In the study by Ponsford et al. (13), patients expressed positive opinions regarding fSCIG, especially a lower disease burden. There was also a reduction in the frequency of injections required for fSCIG compared to SCIG, and similar to our results, good tolerability of the infusion speed was reported. Moreover, all 14 patients in the study by Ponsford et al. (13) experienced temporary side effects at the injection site during infusion. We also found patients reported swelling lasting up to 72 h post-infusion.

Our study presents real-life data from a Polish setting. Currently in Poland, all IgG application modes (IVIG, conventional SCIG, and fSCIG) are available and are reimbursed by the National Health Fund (15). The previous report describing a Polish cohort of 77 adult patients with CVID treated in four centers was published in 2017 (3). Over the follow-up period, over 70% of patients (55/74) changed the therapy mode, mainly from IVIG to SCIG or fSCIG, either directly (IVIG to fSCIG) or indirectly (IVIG to SCIG and then to fSCIG), which was determined mainly by the availability of treatment methods at that time, not the patient's preference itself. The method of switching from IVIG to fSCIG seems preferable for patients previously treated with IVIG every 4 weeks. Indeed, 23 patients in our study refused a conventional SCIG treatment because of the need to administer the drug every 1/2 weeks.

Real-world evidence of fSCIG treatment is limited to case reports of PID (8–11), which are mainly complicated by other medical conditions and require fSCIG introduction due to medical needs. Indeed, in our study, such medical needs included: problems with venous access, adverse events on previous treatment, severe PID complications combined with previous treatment insufficiency, and lack of adherence to previous treatment.

Carne et al. (8) presented the longest (60 months) case of continuous home fSCIG therapy, covering 240 infusions. The patient started on the SCIG method, but trough IgG levels could not be maintained despite high IgG doses (>1 g/kg/month). A change to SCIG facilitated by ovine hyaluronidase was introduced (as at the time, no licensed product containing human recombinant hyaluronidase was available), which normalized IgG trough levels (8). Pedini et al. (9) reported four cases of patients with CVID and accompanying cytopenia, including idiopathic thrombocytopenic purpura (ITP; *n* = 3) and autoimmune hemolytic anemia (AIHA; *n* = 1), who were treated with fSCIG. All patients achieved stable remissions from cytopenia and anemia, and the patient with AIHA was able to switch to a minimal prednisone dose (9).

Danieli et al. (10) reported five cases of patients with PID with different comorbidities who were successfully treated with fSCIG. To achieve satisfactory IgG trough levels and reduce infection rates, a dosing regimen of every 2–3 weeks was used. Similarly, in our study, we found patients with a complex case of PID required more frequent dosing of every 2/3 weeks. Despite this, we found the most common reason for increasing the dose frequency was a wear-off effect, which is often a problem among patients treated with IVIG (24). However, as prior pharmacokinetic analyses indicate fSCIG has a similar pharmacokinetic profile to IVIG (25), such a wear-off effect will likely occur with fSCIG therapy. Therefore, although dosing for fSCIG is designed to be at 3 to 4-weeks intervals (17), clinical practice indicates more frequent dosing every 2 weeks may be needed to achieve the desired effects with good tolerability in certain patients.

Another paper by Wiesik-Szewczyk et al. (11) presents the first case of a successful switch from IVIG to fSCIG during the third trimester of the first pregnancy in a CVID patient. The treatment was effective and well-tolerated by the mother, and provided the baby with sufficient IgG levels (11). In our study, there were two other pregnancies, including a second pregnancy of the patient described before. Both pregnant patients remained on fSCIG therapy and preferred to switch from monthly to biweekly dosing. From these early observations, fSCIG seems to be a good treatment option for pregnant patients with PID.

Although fSCIG therapy was generally well-tolerated in our study and others (8–11, 13), serious conditions may occur, such as necrosis (12). However, more real-life data on fSCIG are needed with larger patient groups, long follow-ups, and different settings, not only to assess its efficacy and safety, but also patient opinions, convenience, and quality of life.

To conclude, here we report the first Polish observational study on the practical experience of fSCIG administration. The major limitation of our study was its retrospective character and a potential record bias. Nonetheless, based on the results from our study, which included a large number of patients and a long follow-up, we conclude fSCIG is safe and efficient for IgG treatment in a real-life setting. Finally, we proved fSCIG efficacy was maintained because of the personalized approach: using different modes of switching (ramp-up, non-ramp-up, and other) and individualized modification of dosing (every 2, 3, or 4 weeks) based on our long-term follow-up data on the shared-decision model of care.

## Data Availability Statement

The datasets generated for this study are available on request to the corresponding author.

## Ethics Statement

The studies involving human participants were reviewed and approved by the Bioethics Committee of the Military Institute of Medicine in Warsaw (Approval No. 55/WIM/2019). The patients/participants provided their written informed consent to participate in this study.

## Author Contributions

EW-S and DS conceived the idea for the study, contributed to the design of the research, involved in data collection, and coordinated the project. EW-S, DS, LP, and KJ-R analyzed the data. All authors have read and approved the final version of the manuscript.

## Conflict of Interest

EW-S and KJ-R received lecture, congress, and advisory board fees from CSL Behring, Kedrion, Octapharma, and Baxter/Baxalta/Shire/Takeda. DS received conference attendance sponsorship from Astellas, Baxter/Baxalta/Shire/Takeda, CSL Behring, Kedrion, Novartis, Octapharma, and Roche; as well as lecturer fees from Baxter/Baxalta/Shire/Takeda, CSL Behring, Kedrion, and Octapharma. The remaining author declares that the research was conducted in the absence of any commercial or financial relationships that could be construed as a potential conflict of interest.
