# Supplementary material for: Facilitated Subcutaneous Immunoglobulin Replacement Therapy in Clinical Practice: A Two Center, Long-Term Retrospective Observation in Adults With Primary Immunodeficiencies
Source: Front Immunol. 2020 May 20;11:981. doi: 10.3389/fimmu.2020.00981 (PMC7326142; doi:10.3389/fimmu.2020.00981)
Supplement: Supplementary file 1 [file Table_1.DOCX]

Supplementary Material

**Supplementary Figure 1.** Comparison of median volumes of infusion (mL) in groups of patients defined by the different infusion intervals (i.e. every 2, 3 or 4 weeks)

(mL)

**Supplementary Figure 2.** Comparison of median IgG through levels (g/L) at different time points
